# Progress in Nanoporous Templates: Beyond Anodic Aluminum Oxide and Towards Functional Complex Materials

**DOI:** 10.3390/ma12162535

**Published:** 2019-08-09

**Authors:** Zimu Zhou, Stephen S. Nonnenmann

**Affiliations:** Department of Mechanical and Industrial Engineering, University of Massachusetts-Amherst, Amherst, MA 01003, USA

**Keywords:** functional oxides, anodic aluminum oxide, nanotechnology, nanomaterials

## Abstract

Successful synthesis of ordered porous, multi-component complex materials requires a series of coordinated processes, typically including fabrication of a master template, deposition of materials within the pores to form a negative structure, and a third deposition or etching process to create the final, functional template. Translating the utility and the simplicity of the ordered nanoporous geometry of binary oxide templates to those comprising complex functional oxides used in energy, electronic, and biology applications has been met with numerous critical challenges. This review surveys the current state of commonly used complex material nanoporous template synthesis techniques derived from the base anodic aluminum oxide (AAO) geometry.

## 1. Introduction

The use of template-based synthetic processes has rapidly grown over the last two decades due to their relative simplicity compared to expensive lithographic approaches and effectiveness in producing scalable 1-D and 0-D nanostructure arrays. Porous membranes such as anodized metal oxide templates [1,2,3], mesoporous carbon [4], zeolites [5], polymer templates [6,7], and nanochannel arrays on glass [8] have been employed in biomedical, optoelectronic, and sensing applications. The majority of templates fabricated comprise binary metal oxides due to their high thermal stability, mechanical compliance, relative abundance, and facile fabrication method. When the alumina self-organized mechanism was first reported by Keller more than 60 years ago, anodic aluminum oxide (AAO) received immediate interest as a means to synthesize nanoscale structures and devices [9]. During a single-step anodization, the pores develop randomly on the oxide surface, limiting their use as a template. The electrochemical oxidation of transition metals, including hafnium (Hf) [1,10], tantalum (Ta) [11,12,13,14], and titanium (Ti) [15,16,17], also produce a porous thin film structure on their surfaces. Older studies that achieved ordered nanostructures required time/cost intensive methods such as focused ion beam (FIB) milling, scanning probe scraping, or hard SiC/Si_3_N_4_ molding to produce a pre-pattern step on the surface [18,19,20]. In the mid-1990s, Masuda and Fukuda overcame this challenge by pioneering a two-step anodization process in an acidic electrolyte that produced an aluminum oxide thin film that possessed a uniform nanoporous internal structure [21]. Unlike conventional lithographic processes, this inexpensive method enables scalable formation of a periodic pore network over a relatively large area by adjusting parameters such as applied voltage, temperature, and electrolyte selection.

Here, we review a new direction in nanoporous template production—translating the ordered porous arrays found in binary oxides to more complex, multi-component materials that are unable to be patterned by conventional anodization methods. This review begins with a brief background and survey of anodic aluminum oxide (AAO), the most commonly used nanoporous template for 1D and 2D nanomaterials fabrication. The scope of nanoporous templates is then extended to include transition metal oxides and alloy oxides. These metallic-derived templates use the same AAO anodization technique but possess more inherent functionality than a wide-gap insulator such as alumina. In the second half of the review, the focus shifts to highlighting recent developments of complex compound porous templates requiring the use of AAO reverse templates, a nanoporous template that mirrors the porous network of AAO yet comprises multicomponent, functional materials such as carbides, nitrides, and perovskite (ABO_3_) oxides.

## 2. Anodic Aluminum Oxide (AAO) and Its Applications

The AAO fabrication process enables tailoring of pore diameter (D_pore_), interpore distance (D_int_) and oxide layer thickness by controlling parameters such as anodizing voltage, temperature, and electrolyte type. For a two-step anodization, the high purity aluminum undergoes an initial anodization step followed by a chemical etching step (typically with chromic/phosphoric acid) that selectively removes the newly formed yet irregularly patterned aluminum oxide layer without damaging the aluminum substrate, resulting in an ordered and textured surface available for a second anodization. This textured surface creates accurate pore growth positions during the second anodization, since pore nucleation preferentially occurs at surface defects. The second anodization is usually carried out under the same condition as the first step. The duration of this second anodization process controls the final thickness of the AAO membrane. The AAO geometric structure is also adjustable by post-anodization processing. Immersing the template in dilute phosphoric acid at room temperature enlarges the AAO pore size without changing the interpore distance [22,23,24]. Conversely, conformal Al_2_O_3_ coatings through atomic layer deposition (ALD), a method with high uniformity and thickness control [25,26,27], is capable of narrowing the pore diameter down to 5 nm [28]. For more details concerning conventional AAO fabrication technique and its direct applications in nanomaterials, we suggest reading some excellent review articles on AAO fundamental principles, techniques, and applications [18,29,30,31,32].

### 2.1. AAO Template Assisted Fabrication of Nanodots Arrays

Aluminum anodization is a cost-effective, highly-controllable, and easily scalable process that produces an ordered array of vertical channels that have been used extensively for the fabrication and the synthesis of nanostructures and devices ranging from 0-D (nanodots, nanoislands) to 1-D (nanowires, nanotubes). Ultrathin AAO templates, defined as those possessing an aspect ratio (pore depth to pore diameter ratio) of less than 10, can be separated from its host aluminum substrate and transferred to a second, arbitrary (functional) substrate when reinforced with a polymer coating such as poly (methyl methacrylate) (PMMA) or polystyrene (PS) that assists with mechanical compliance.

AAO-directed fabrication of ordered 40 nm Au nanodot arrays on a silicon substrate were among the first templated synthetic studies conducted in the late 1990s [33]. This method circumvented the need for costly electron beam lithography, which typically suffers from low throughput due to its prolonged exposure time. Other metals such as Au, Ni, Co, and Fe have also been deposited on a variety of substrates using this approach [34,35]. Recent studies that employed ultra-low aspect ratio membranes (1:2) extended this approach to achieve nanoisland diameters down to 16 nm [36]. High-density nanoisland arrays have also be extensively studied as an ideal architecture for functional oxides, which exhibit remarkably different properties than conventional bulk materials.

**Figure 1:**
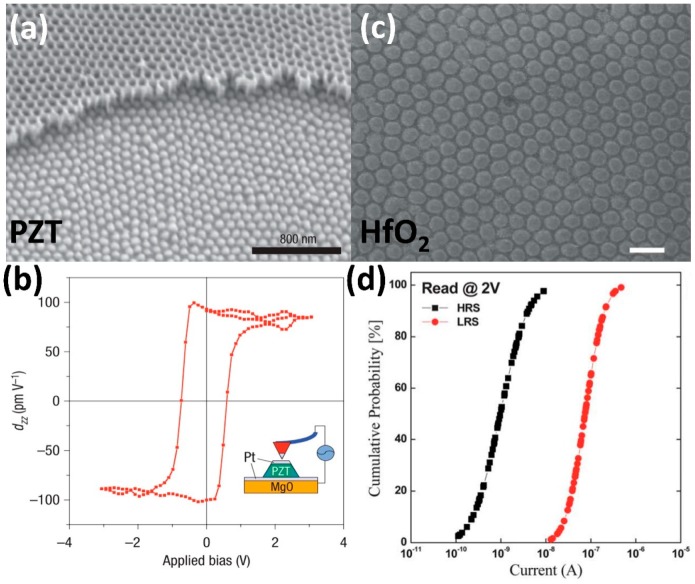
(**a**,**b**). Individually switchable PZT nanodots arrays partially covered by anodic aluminum oxide (AAO) mask and its piezoelectric hysteresis loop, reproduced with permission from Ref. [37], 2008 Nature Publishing; (**c**). HfO_2_ nanodots with conductive substrate (200 nm scale bar); (**d**). Cumulative probability plot obtained by 65 I–V cycles at reading bias of 2 V, reproduced with permission from Ref. [25], 2012, RSC Publishing.

The pioneering work of Lee et al. reported on the ultrathin, template-directed fabrication of a versatile metal/oxide/metal (M-O-M) (Pt/PZT/Pt) nanoisland nanocapacitor structure deposited on an MgO single crystal substrate (Figure 1a) [37]. The isolated pattern localized the electric field distribution, minimized the effective cross-talk between structures, and enabled each nanocapacitor to be individually addressable (Figure 1b), thus the array exhibited an extremely high storage density (176 Gb inch^−2^) [37]. Template-directed fabrication of nanocapacitors comprising functional materials such as HfO_2_ (Figure 1c) and Ag_2_S (not shown) have been used to form nanoscale memristive cells, which show controllable resistive switching (RS) properties (Figure 1d) suitable for nonvolatile memory applications [25]. The combination of highly tunable, scalable templates and thin film deposition creates large arrays of M-O-M nanocapacitors, thus becoming one of the most precise and convenient methods in nanomaterials test structure fabrication. Other studies deposited cobalt ferrite oxide (CFO) nanodots epitaxially through ultrathin, template-directed pulsed layer deposition (PLD). By tuning the anodization conditions, the magnetic behaviors of nanodots (60–300 nm diameter and 60–500 nm interpore distance) were observed via magnetic force microscopy (MFM), with clusters comprising smaller diameter nanodots exhibiting dipole–dipole interactions of opposite phase and larger diameter nanodots possessing opposing phases within a single structure [38].

### 2.2. AAO Template Assisted Fabrication 1-D Nanostructures

One dimensional nanostructures such as nanorods, nanowires, and nanotubes demonstrate enormous potential in the fields of magnetic, electronic, and optoelectronic devices. Nanorods possess an aspect ratio of less than 10, while nanowires are defined by an extremely high aspect ratio, usually exceeding 1000. The high degree of controllability of AAO dimensions, including accurate control of nanowire length and diameter, motivates the use of AAO-template-assisted approaches such as electrodeposition and sol-gel as attractive alternatives to multistep lithographic methods.

**Figure 2:**
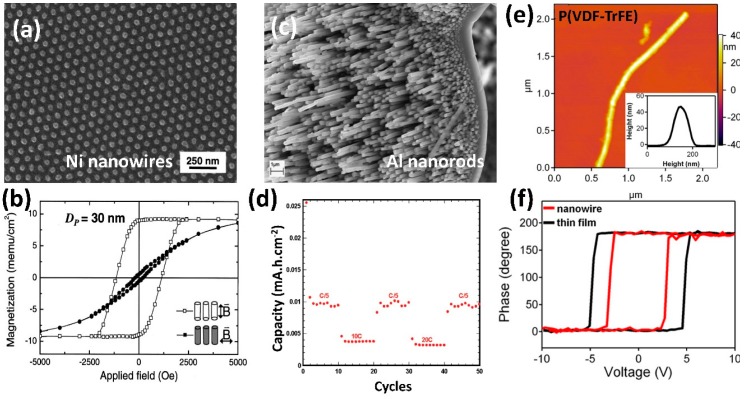
(**a**) SEM image of Ni-filled AAO template; (**b**) hysteresis loops for Ni nanowires with diameters of 30 nm from Ref. [39], 2001 AIP Publishing; (**c**) oblique view of aluminum nanorods reproduced with permission from Ref. [40], 2008, Elsevier Publishing; (**d**) 50 cycles experiment of Al nanorods with TiO_2_ coating from Ref. [41], 2009 American Chemical Society; (**e**) An atomic force microscopy (AFM) topographic image of poly(vinylidene fluoride with trifluoroethylene [P(VDF-TrFE)] nanowire on silicon substrate and (**f**) A piezoresponse force microscopy (PFM) phase hysteresis loop on a 60 nm diameter P(VDF-TrFE) nanowire (red) and a 60 nm thin film (black) from Ref. [43], 2013 American Chemical Society.

Examples include high density nickel (Ni) nanowires arrays (Figure 2a), which exhibited an increase in magnetic coercivity with decreasing Ni nanowire diameter and subsequently improved magnetic hardness (Figure 2b) [39]. Each nanowire was capable of being switched independently, thus producing a recording density up to 155 Mbit/mm^2^ for Ni nanowire array. This density was significantly higher than the density within currently available hard drives (5.74 Mbit/mm^2^) [31]. One-dimensional nanostructures inherently possess high surface-to-volume ratios, motivating their use as next generation power source electrodes and microbatteries. Freestanding aluminum nanorod electrode arrays were produced by template-directed electrodeposition (Figure 2c) [40] and were subsequently coated with a thin layer of titanium dioxide (TiO_2_) via atomic layer deposition (ALD), which resulted in an increase of the overall capacity by an order of magnitude and the stability for over 50 charge/discharge cycles (Figure 2d) [41].

AAO templates also assist the synthesis of low melting point metals, semiconductors, and functional polymers nanowires by vacuum melting and solution wetting. Zhang et al. melted bismuth (Bi) into an AAO template at 325 °C (T_m_ = 271.5 °C) in vacuum and introduced high pressure argon to inject liquid bismuth into the nanopores, resulting in an extremely high area density (7 × 10^10^ cm^−2^) array of individual, single crystal nanowires with strong implications for thermoelectric applications [42]. Other studies demonstrated the preparation of ferroelectric poly(vinylidene fluoride with trifluoroethylene [P(VDF-TrFE)] nanowires with the diameter of 40–80 nm by solution wetting in AAO templates (Figure 2e) [43]. Due to the geometric constraint within AAO nanochannels, the calculated coercive field (switching voltage/thickness) of the released nanowire was found to be ~40 MV/m, which was significantly lower than its bulk (~50 MV/m) and thin film (~80 MV/m) counterparts (Figure 2f) [44]. Another simple but innovative method was introduced to synthesize palladium (Pd) nanotubes, where AAO templates were first wetted by poly (D,L-lactide) (PDLLA) and palladium acetate [Pd(OAc)_2_] and then dried to form solid PLDDA/Pd(OAc)_2_ nanotubes [45]. Annealing the structures for varying time periods produced pure polycrystalline Pd nanotubes with different wall morphologies. Pd nanotubes of controllable dimensions and morphology are of interest for applications such as fast-response hydrogen sensors [46] and highly-efficient catalysis [47]. Due to its inherently porous nature and biocompatibility, AAO is also widely applied in biochemical fields such as drug delivery, tissue engineering, and biocapsules [48,49,50,51].

## 3. Transition Metal Oxides and Alloy Templates

### 3.1. Single-Component Transition Metal Oxides Template

**Figure 3:**
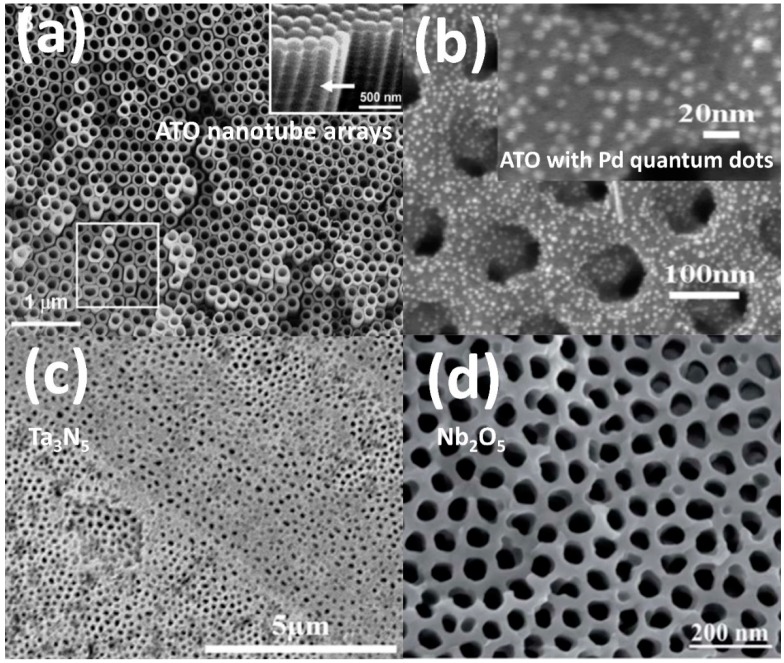
(**a**). Typical anodic titanium oxide (ATO) nanotube arrays from Ref. [3], 2008 Wiley-VCH; (**b**). Pd quantum dots deposited on ATO arrays from Ref. [64], 2012 American Chemical Society; (**c**) SEM image of Ta_3_N_5_ nanotube arrays from Ref. [13], 2015 ELSEVIER; (**d**) SEM image of blackened Nb_2_O_5_ nanochannels from Ref. [54], 2015 Royal Society of Chemistry.

The enhanced surface area of template-fabricated 0-D and 1-D nanostructures motivates their use in gas sensing [15,52], photovoltaic [53], and water splitting [13,14,54,55] applications, among others. Over the past 20 years, attempts to extend template fabrication beyond Al_2_O_3_ to construct nanoporous networks in other functional (binary) metal oxides have progressed rapidly. Since the discovery of self-organized pores within TiO_2_ in 1999 [56], advanced anodization methods have been applied to a wide variety of transition metals. Unlike AAO, which comprises a continuous porous network, anodic titanium oxide (ATO) templates consist of individual nanotubes produced by a one-step anodization process (Figure 3a) [3]. Annealing the amorphous, as-grown ATO template converts it into its crystalline anatase form, which possesses a higher charge carrier mobility [57]. ATO nanotubes possess a large surface area and a short diffusion pathway and thus attract considerable interest as anodes in dye-sensitized solar cells (DSSCs), which have been extensively studied as an energy harvester due to their excellent light to electricity conversion efficiency [16,17,58,59,60].

A modern DSSC assembly typically comprises a porous, microns-thick TiO_2_ anode, where the efficiency *η* is heavily influenced by dye concentration, oxide structure, and surface area [61], resulting in efficiencies higher than commercial nanostructured titanate photocatalyst [62,63]. The photocatalytic activity is further enhanced through the deposition of noble metal nanoparticles over the nanotubular structure surface (Figure 3b) [64,65,66], which produces a high conversion efficiency under full sunlight and low-loss liftetimes (>1000 h) under accelerated thermal stress tests [67]. ATO templates possessing smaller pore diameters (22 nm) exhibited a 10^4^ electrical conductivity increase exposed to hydrogen at 290 °C, an order of magnitude higher than previously reported hydrogen sensitivity values [68]. Conversely, large diameter, lower surface area ATO (76 nm) exhibited a hydrogen sensitivity reduction by a factor of 200. Here, the surface available for the chemisorption of hydrogen spillover yields an electron accumulation inside the nanotube that increases electrical conductivity [3].

Tantalum pentoxide (Ta_2_O_5_) is a high-κ dielectric used in a wide range of applications such as dielectric layers for storage capacitors [69], implant coatings [11], as well as an efficient photocatalyst for water decomposition [70]. Anodization and annealing processes of high purity tantalum create a facile fabrication route for Ta_2_O_5_ semiconductor nanotube arrays. As one of the most stable transition metals, Ta-based Ta_2_O_5_ can withstand ultra-high temperatures without compromising its microstructure. Recent studies showed that annealing Ta_2_O_5_ nanotube (NT) arrays at 800 °C between 30 to 60 min tripled the crystallinity and nearly doubled the hydrogen photoproduction rate [14]. Ta_2_O_5_ possesses a wide band gap (*E_g_* ~ 4 eV) that severely limits its solar energy harvesting ability [71]. Recent efforts have converted Ta_2_O_5_ templates into Ta_3_N_5_ under a constant ammonia flow at 1000 °C, resulting in an NT array structure (Figure 3c) with a significantly reduced band gap of 2.1 eV [13]. The as-prepared undoped Ta_3_N_5_ photoanode decorated with Co-Pt co-catalysts were claimed to have achieved the highest photocurrent (5.9 mA/cm^2^ at 1.23 V_RHE_) at the time of publication. Niobium oxides (Nb_2_O_5_) NT arrays have also been used for photoelectrochemical water-splitting. Based on the processes developed for blackened TiO_2,_ which significantly increases the band gap and the solar absorption [72], studies of an oxygen-deficient black Nb_2_O_5_ template (Figure 3d) showed an extremely broad absorption spectra spanning from the UV region to the IR region and exhibited a highly enhanced 1.02 mA/cm^2^ photocurrent, making reduced niobia a promising candidate for photoelectrochemical (PEC) photoanode material [54].

### 3.2. Multi-Component Transition Metal Templates

Due to the nature of self-organization, nanoporous binary oxide templates result from competing chemical dissolution and electrochemical formation processes [30]. Use of a single type of metal limits the geometric degrees of freedom and thus yields a hierarchically ordered structure. As shown by the various examples above, materials functionality is greatly enhanced by the multi-scale features of the nanoporous template. Such an approach motivated exploration into the synthesis of multicomponent oxides by the anodization of alloys. The anodization of Group IVB and VB valve metal alloys has two main advantages: (1) biocompatibility—transition metal alloys have been widely studied and used for implants due to their superior biocompatibility and corrosion resistance compared to their (individual) metallic constituents [73,74,75]; (2) transition metal-like properties— anodization ideally maintains balance between chemical dissolution and oxide formation. The chemical properties of transition metals minimize two major problems hindering common alloy anodization: *(i)* different dissolution rates of the constituent elements and *(ii)* varying reaction rates in different alloy phases. This unique feature ensures the uniformity of self-organization during anodization of binary oxides.

**Figure 4:**
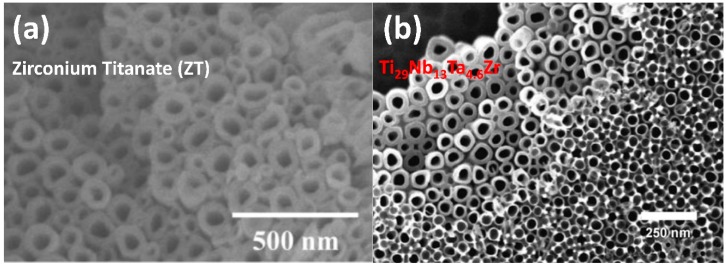
(**a**) SEM image of Ti-Zr alloy arrays with ~90 nm diameter and ~30 nm wall thickness, reproduced with permission from Ref. [76], 2007 Wiley-OCH; (**b**) alloy nanotubes broken off at different height levels from Ref. [79], 2006 Wiley-VCH.

Yasuda demonstrated excellent structural flexibility and controllability of alloy oxides nanotube by tuning the zirconium titanate (ZT) nanotube arrays morphology (Figure 4a) [76,77]. Pure zirconia nanotubes processed by conventional methods possessed a nominal diameter of 50 nm and a length of 17 µm [78], while pure titanium oxides nanotubes possessed a nominal diameter of 100 nm and a length of 2.5 µm [17]. When anodizing a 50:50 wt.% Ti-Zr alloy in 1M (NH_4_)SO_4_ + 0.5 wt.% NH_4_F solution, the oxides matrix showed significantly higher controllability of the structure; the pore diameter ranged from 15 to 470 nm and the length up to 21 µm, depending on the anodization conditions. Anodization of the single-phase metal alloy Ti_29_Nb_13_Ta_4.6_Zr in a fluoride-based electrolyte produced a unique two-scale pore diameter (d = 32 nm; 55 nm) within a single oxide porous template (Figure 4b) [79]. Auger electron spectroscopy (AES) determined that the tantalum oxide composition was increased in both 32 nm and 55 nm nanotubes in comparison with other metal oxides throughout the alloy oxides layer, which proved the higher chemical dissolution rate of the other metal oxides. This coincides with the fact that Ta_2_O_5_ has the highest chemical stability among all other transitional metal oxides, which means it has the lowest chemical dissolution rate [12]. The results demonstrate even subtle changes in alloy chemical composition can induce large variations in oxide formation, thus motivating new anodization processing directions for multi-scale porous template fabrication.

**Figure 5:**
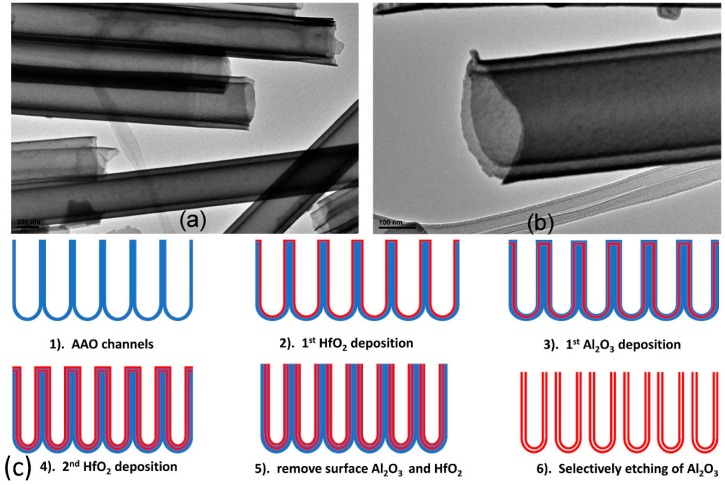
(**a**,**b**). TEM cross-sectional images of released coaxial HfO_2_ nanotube; (**c**) (**1**) pre-engineered AAO channels, (**2**) first deposition of HfO_2_ layer, (**3**) deposition of Al_2_O_3_ layer, (**4**) second HfO_2_ layer deposition, (**5**) removal of surface HfO_2_ and Al_2_O_3_ layer to expose Al_2_O_3_ layers by ion milling, (**6**) selective etching of Al_2_O_3_ sacrificial layers by NaOH, leaving separated coaxial nanotubes. Reproduced with permission from Ref. [80]. Copyright 2010 American Chemical Society.

The tunable wall thickness of nanotubes provides one degree of freedom more than nanowires possess. A novel layer-by-layer deposition approach leveraged this design flexibility to create a nested, multiple-walled coaxial binary oxide nanotube structure (Figure 5a,b) [80], where the first binary oxide layer was precisely and uniformly deposited on an AAO template sacrificial base (Figure 5c-i) by atomic layer deposition (ALD) with angstrom-level resolution (Figure 5c-ii). A second, sacrificial Al_2_O_3_ layer and a binary oxide layer were deposited using the same strategy (Figure 5c-iii,iv). The extra sacrificial Al_2_O_3_ layer serves as an insulator that effectively separates different oxide layers. The top surface of the as-grown multi-layered sample was polished by ion milling to expose the AAO layer (Figure 5c-v) for selective etching of the alumina layers (Figure 5c-vi) using a 1 M NaOH solution. The enhanced surface area of the (hollowed) multi-layer nanotube arrays allows additional parametric freedom in their design. This unique feature could facilitate fundamental studies such as complex biosensors or photovoltaic devices or magnetization reversal phenomena related to magnetic nanotube wall thickness [81].

## 4. Functional Reverse Template

The well-studied effects of anodization conditions on sample morphology and geometry enables fine tuning of pore size, pore density, and channel length of oxide templates. Routine production of pore diameters ranging from 20 nm to 500 nm and aspect ratios from <10 to 1000 s yield a robust, versatile platform to explore size effects within nanoscale materials. The preceding sections highlighted three primary advantages of using oxides templates: (1) template-directed nanostructure deposition—due to their highly uniform, periodic porous structure, oxide templates enable top-down, size-controlled fabrication of electrochemically deposited or sputtered/evaporated metal and metal-oxide nanostructures; (2) catalyst supports—various catalytic processes are greatly enhanced due to the extremely large surface area provided by oxide templates; (3) inexpensive, facile fabrication—uniform nanoscale features are easily reproduced without using traditional expensive lithographic techniques. Applications utilizing AAO templates, however, are restricted by the limited, inherent functionality of alumina or other binary oxides.

From an application standpoint, developing 2D nanoporous arrays in complex oxides confers functional properties that extend beyond AAO templates to include ferroic behavior, defect-mediated memristive switching, and biocompatibility, among many others. Due to their chemical stability, fabrication of 2D templates comprising complex oxides and noble metals must use conventional methods such as lithography and nanoimprinting. These expensive, time-consuming techniques severely hinder the development and the application of functional oxide templates. Thus, 2D functional material template fabrication requires new template-assisted methods to create ordered, nanoporous arrays. The so-called 2D anti-dot nanostructure remains one underexplored yet viable option towards achieving these nanoporous arrays. Three major anti-dot array fabrication methods exist: (1) direct deposition; (2) plasma etching; and (3) reverse replica fabrication.

### 4.1. Direct Deposition: Magnetic Storage Media

Direct deposition uses the engineered AAO template of a chosen geometry and dimensions as the substrate for conventional deposition methods such as sputtering, ALD, and thermal evaporation [82,83,84]. Well-developed deposition techniques are able to control the deposition thickness on the Angstrom (Å) level and thus preserve the original morphology of the AAO template [26,80]. Porous magnetic structures have been extensively studied in the last decade due to their potential as ultra-high density magnetic storage media and the rich, fundamental physics underlying their operation [85,86,87]. As compared to individual nanodots, anti-dot arrays generally display two advantages: *(i)* superparamagnetism does not exist nor hinder the data-storage unit size, since the storage device is a continuous film [88], and *(ii)* coercivity and remanence can be controlled by varying anti-dot pore size [89]. However, most studies utilize inefficient traditional patterning approaches such as block co-polymer templating and lithography. Expensive fabrication equipment, small deposition areas, and relatively large feature sizes (200 nm to 400 nm) all limit the use of these techniques in producing next-generation anti-dot device arrays [86,87]. AAO templates represent a viable alternative due to easily tunable pore diameters below 50 nm. AAO-produced magnetic 2D nanostructures have displayed a storage density of 1 Tb in^−2^ [90], motivating further miniaturizing of magnetic storage components and creating a competitive candidate system for future high-density magnetic data storage devices.

**Figure 6:**
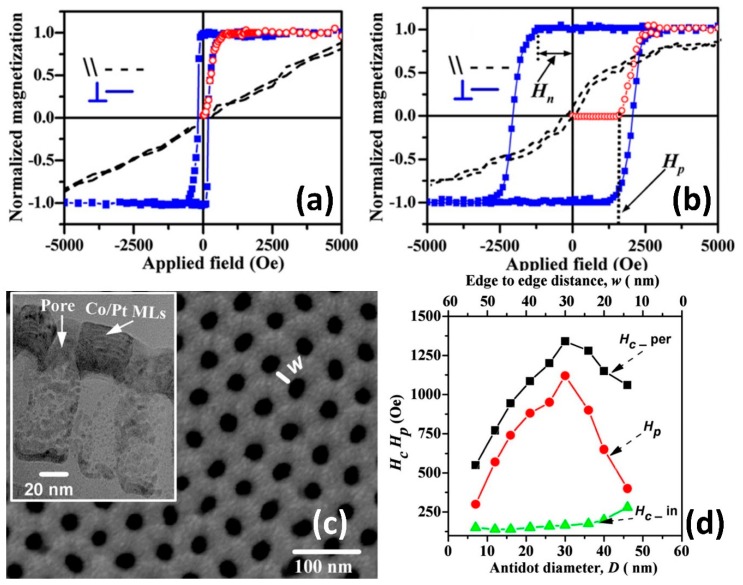
(**a**) Out-of-plane and in-plane hysteresis loop of continuous Co/Pt thin film and (**b**) anti-dot arrays with average pore diameter of 9 nm. Reproduced with permission from Ref. [90], 2008 IOP Publishing; (**c**) SEM image of Co/Pt anti-dot arrays with 25 nm diameter and 35 nm interpore distance. Inset of TEM cross-sectional image and (**d**) magnetization dependence on anti-dot morphology from Ref. [82], 2010 APS Publishing.

Rahman et al. thoroughly investigated the dependence of magnetization on anti-dot morphology by comparing the perpendicular coercivity (H_c_) of continuous and porous film (Figure 6a,b) [90]. The study utilized an AAO template with a ~9 nm nominal pore size that was used as the base substrate, which was subsequently sputtered with 0.5 nm thick Co and 2 nm thick Pt films. The as-deposited magnetic anti-dot arrays exhibited an extremely high density of 2.1 × 10^11^ cm^−2^, a squareness ratio (of remnant magnetization to saturation magnetization) of unity, and negative nucleation fields, which are the preferred features for high density recording devices [90]. Another study sputtered a thin Co/Pt film on an AAO template to produce magnetic anti-dot arrays with a pore size range between 7 and 46 nm (Figure 6c), of which the 30 nm (Figure 6d) anti-dot array exhibited the highest out-of-plane coercive field *H_c_* = 1350 Oe and out-of-plane magnetic hysteresis loop squareness ratio S [82]. Continuous films only exhibited a coercive field of 140 Oe with negligible squareness (Figure 6d, green line), while porous films with larger pore sizes displayed sharp reductions in H_c_ (Figure 6d, red line) due to high anisotropy in the vicinity of the pore rim [91]. The enhanced coercivity is attributed to the anti-dot array serving as non-magnetic defects, which effectively results in stronger domain-wall pinning with increasing pore size [92]. Interestingly, pinning fields decreased as anti-dot size exceeded 30 nm due to the rim of the pores having the freedom to tilt towards the surface and induce magnetization tilting. Albrecht et al. observed similar behavior manifesting as variations in multilayer Co/Pd nanosphere anisotropy directions [93]. While vacuum deposition techniques can routinely produce complex oxides thin films with other functionalities such as resistive switching and ferroelectricity, deposition of ultra-thin films on AAO surfaces to produce anti-dot arrays have been mostly limited to magnetic metals, as the deposited thin film cannot be separated from the AAO template. This renders alumina, an electrical insulator, as a poor substrate for developing modern metal-insulator-metal (MIM) structured nanocapacitors.

### 4.2. Dry Etching: Multiferroic Bismuth Ferrite Anti-Dot Arrays

**Figure 7:**
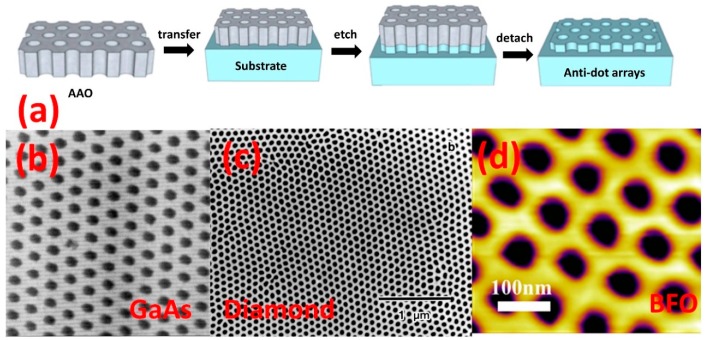
(**a**) Schematics of etching method procedure. Reproduced with permission from Ref. [30], 2014 American Chemical Society; (**b**) AFM image of GaAs nanoporous thin film with 60 nm diameter pores from Ref. [98], Copyright 1999 the Japan Society of Applied Physics; (**c**) SEM image of diamond anti-dot arrays from Ref. [95], 2000 Wiley-VCH; (**d**) topography of anti-dot BiFeO_3_ (BFO) arrays. Reproduced with permission from Ref. [104], 2016 IOP Publishing.

Combining conventional AAO-directed fabrication with top-down ion etching methods allows the user to separate film fabrication from morphology modification, resulting in a more flexible operating parameter window while improving thin film quality. In this process, a pre-engineered through-hole AAO serves as a nanostructured mask (Figure 7a-i) as transferred on a desired substrate (Figure 7a-ii) comprising a functional material, either a metal or metal oxide. Then, ordered nanohole arrays are fabricated by conventional dry etching processes such as ion milling, reactive ion etching (RIE), and plasma etching (Figure 7a-iii,iv) [30,34,94,95,96,97,98,99,100,101,102,103,104]. Due to its high tolerance to oxygen etching, AAO makes an ideal material for high aspect ratio etching masks. In 1999, the Masuda group first translated the highly ordered nanochannel structures of AAO into III-V semiconductors (GaAs and InP) by using reactive beam etching (RBE) [98] (Figure 7b). Their group also used oxygen plasma etching to form ordered diamond sub-100 nm anti-dot arrays over 1 cm^2^ [95], an area which would be extremely time-consuming to cover by traditional lithography methods (Figure 7c). Nanoporous diamond thin films are promising candidates for future high performance nanocapacitors due to high charge per unit capacitance ratios that exceed those of graphitic carbons [99].

Tian et al. extended this method to complex oxides field by synthesizing BiFeO_3_ (BFO) anti-dot arrays [104]. Here, thin layers of SrRuO_3_ (SRO) and BFO were first grown epitaxially on SrTiO_3_ (STO) by pulsed layer deposition (PLD), and then an ultra-thin (aspect ratio < 8) AAO membrane was transferred to the substrate as a morphology modification mask. Subsequently, the as-prepared sample was etched by an Ar^+^ ion beam followed by mechanical removal of the AAO. Variation in the etch duration produced differently ordered nanostructure morphologies: nanorings (5 min), anti-dots (10 min, Figure 7d [104]), nanochains (20 min), and nanodots (25 min). This efficient, precise approach is also exploited in the fields of advanced optoelectronic devices [100], photonic bandgap waveguides [94], and anti-reflection coatings [101]. However, as AAO is also removed during the etching process, its pore size is continuously increasing while its thickness is decreasing, thus structural controllability is always an issue. Overall, proper balance between the AAO mask feature size, the AAO thickness, and the final feature size in the target oxide is critical to successful anti-dot formation.

### 4.3. Negative Replica Method

**Figure 8:**
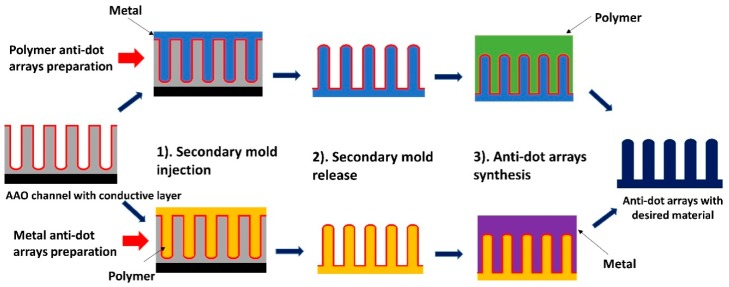
Schematics of anti-dot arrays fabrication. Starting material is AAO templates coated with a thin conductive layer. (**1**) Secondary mold injection. Metal secondary mold can be electrochemically deposited into channels while polymer mold spontaneously covers the channels. (**2**) Free standing secondary mold is obtained by selective etching of AAO template. (**3**) Polymer/metal antidot arrays are formed by wetting or electrochemical deposition, respectively.

A negative replica template is a secondary mold with a nanopillar surface structure that mirrors the original AAO tunnel structure. Polymer solutions with lower surface energy than aluminum oxide tend to spontaneously wet the inner surface of the AAO channels [105]. Thus, high performance polymers are frequently chosen as the secondary mold material, since they can be directly injected into template channels to form a negative replica. The desired complex oxide, nitride, or carbide is subsequently deposited evenly on the negative mold surface, which forms the duplicate of the original AAO master porous structure. The final template with structures identical to the AAO master is then obtained by selectively removing the secondary mold. Masuda formed functional replica membranes of Pt [17], Au [17], Ni [106], and CdS [107] by injecting PMMA as a sacrificial secondary mold. Nanoporous polymeric replicas can also be obtained by using metallic secondary molds (Figure 8) [108,109]. The thickness of metallic replicas is limited by the ability to form polymer secondary replicas with high aspect ratios. To address this limitation, one innovative study electrochemically deposited the mold materials to the side of AAO instead of infiltrating the secondary mold into the AAO channels, thus providing sufficient mechanical support to keep the PMMA nanopillars upright. The resulting Ni reverse template achieved a high aspect ratio of 20.

**Figure 9:**
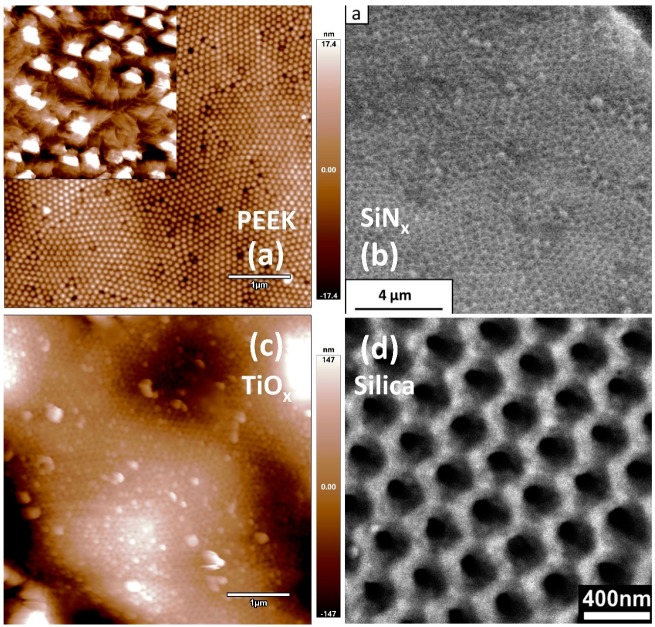
(**a**) AFM image of poly (ether-ether-ketone) (PEEK) nanorod arrays and (**inset)** PEEK nanorods aggregate in bundles when aspect ratio exceeds five; (**b**) SEM image of SiN_x_ anti-dot arrays. Reproduced with permission from Ref. [111], 2012 RSC publishing; (**c**) TiO_2_ anti-dot arrays; (**d**) SEM image of Silica replica. Reproduced with permission from Ref. [113], 2008 American Chemical Society.

The negative replica synthetic method is amenable to processing an array of commercially available high-performance polymers such as poly (ether-ether-ketone) (PEEK) and poly(tetrafluoroethylene) (PTFE), which are extremely difficult to modify using conventional methods [30]. Pellets of the selected polymer are placed on the top of AAO template and simply melted to infiltrate the AAO pores. As such, virtually all polymer solutions with lower surface energy than aluminum oxide can serve as a negative replica material using the template wetting method. After removing AAO, the polymeric negative replica with reverse AAO nanostructure (positive nanopillars) is subsequently used as a mold to fabricate anti-dot arrays with complex materials (Figure 9a inset). The only disadvantage of this method is that reverse template nanostructures will aggregate into bundles and lose their order (Figure 9a inset) when the aspect ratio exceeds five due to strong capillary forces between nanopillars [110]. For example, Martin et al. melted PEEK (aspect ratio < 6), a highly chemically stable polymer, into the original, engineered AAO template at 390 °C and then selectively dissolved the aluminum substrate and AAO in an acidic solution of CuCl_2_ and NaOH 10 wt.%, respectively, without damaging PEEK nanorod [111]. The self-standing PEEK nanorod template was then taken as a mold for silicon nitride (SiN_x_) deposition and thermally removed at 600 °C. The resulting SiN_x_ template perfectly preserved the original AAO template nanoporous network structure (Figure 9b). The SiN_x_ template displayed many superior properties compared to the original AAO master template, including greater high temperature strength, abrasive resistance, and chemical inertness. Our group used the negative replica method to successfully fabricate a 70 nm pore size through-hole continuous TiO_2_ anti-dot array, where TiO_2_ was sputtered to PEEK nanorod arrays to form a nanoporous structure. The PEEK template was subsequently thermally removed to leave a free-standing TiO_2_ anti-dot array (Figure 9c). This method demonstrates its ability to fabricate complex and functional anti-dot arrays and extend the negative replica sequence to materials beyond binary oxides, considering the advances of vacuum thin film deposition techniques (sputtering, ALD, PLD). 

While the fabrication of the PEEK nanomold is costly and time-consuming, thermal removal of the final nanoporous template is destructive. To reduce the cost of the precursor wetting method, Martin et al. fabricated an ordered, 120 nm diameter nanopillar array of poly(tetrafluoroethylene) (PTFE) that extended over large (cm^2^) areas [112]. The PTFE negative replica was subsequently immersed in a 10 wt.% solution of poly(vinyl alcohol) (PVA), a well-known biocompatible material, and then dried in vacuum to form anti-dot arrays. Since the nanomold was not damaged during the process, the PTFE nanomold could be directly separated from the anti-dot template using tweezers and reused for preparing additional templates, thus increasing throughput and cost-efficiency. Preserving the original ordered nanoporous structure after removing the AAO master template, however, is still a challenging step in template-directed replica synthesis. Also, while most high-performance polymeric materials are chemically robust, their low thermal stability restricts the overall processing temperature window, which is a significant issue, as most complex oxides require high, elevated temperatures to produce the desired (and often functional) phase. Thus, an additional post annealing step is required to functionalize the as-deposited film, which could also potentially damage the nanoporous network.

Another route towards non-destructive replication transferred the AAO nanoporous structure to a silica (SiO_2_) template (Figure 9d) [113]. Here, an amine-modified resin was injected into the AAO channels and exposed to UV light (350–400 nm) to form cross-linked polyacrylate nanofibers inside the pores. The nanofiber arrays were then gently separated from the AAO template by being attached to a piston and lifted with a force around 0.4 N at a rate of 0.1 mm/min. The resulting negative polyacrylate replica array was covered with a sol-gel to form a porous silica template, which was finally detached from the nanofiber arrays using tweezers. The as-prepared nanofiber arrays perfectly replicated the AAO porous structure up to an aspect ratio of 10; higher aspect ratio structures (~30) showed the tendency to bend and agglomerate. This issue could potentially be solved by modifying the AAO template morphology or using higher-strength materials as the negative replica. This non-destructive method enables multiple duplicate templates from a single AAO template by recycling both the AAO template and its negative replica, which greatly reduces cost and labor of the entire process.

## 5. Conclusion

Due to the nature of self-organization in nanopore formation, direct anodization is unable to produce an ordered porous structure in complex materials such as carbides, nitrides, or perovskite oxides. With the help of modern fabrication techniques, self-ordered nanoporous templates have been utilized to bridge the gap between anodized templates and patterned complex materials. We surveyed a selection of direct anodization of binary metal oxides templates and templated assisted fabrication of functional porous templates using methods ranging from ion etching to reverse template replication. Continued improvement in deposition techniques and lab facilities will spawn future synthetic routes towards facile fabrication of complex material templates with flexible dimensions to potentially serve as matrices for advanced nanocomposites to be filled with complementary material pairings to produce enhanced electrical, electrochemical, ferroic, biological, and optoelectronic properties and functionality.
